# Copper(II) hydrogenphosphate, CuHPO_4_

**DOI:** 10.1107/S1600536809045632

**Published:** 2009-11-07

**Authors:** Christiane Günther, Helmar Görls, Dörte Stachel

**Affiliations:** a Otto-Schott-Institut, Friedrich-Schiller-Universität Jena, Frauenhoferstrasse 6, 07745 Jena, Germany; b Institute of Inorganic and Analytical Chemistry, Friedrich-Schiller-Universität Jena, August-Bebel-Strasse 2, D-07743 Jena, Germany

## Abstract

The title compound, CuHPO_4_, has been synthesized from a mixture of phospho­ric acid and copper oxide. It has the same composition as *M*HPO_4_ (*M* = Ca, Ba, Pb, Sr or Sn), but adopts a rhombohedral structure with all atoms on general positions. The structure features distorted PO_4_ tetra­hedra linked by copper, forming 12-membered rings. The Cu^II^ atom is coordinated by five O atoms in a distorted square-pyramidal manner. O—H⋯O hydrogen bonding leads to an additional stabilization of the structure.

## Related literature

For the structure of CaHPO_4_, see: Smith *et al.* (1955 ▶); MacLennan & Beevers (1955 ▶). For a report about BaHPO_4_ and PbHPO_4_, see: Bengtsson (1941 ▶). For the structure of SnHPO_4_, see: Berndt & Lamberg (1971 ▶). For information about SrHPO_4_, see: Boudjada *et al.* (1978 ▶). For a report about CuHPO_4_·H_2_O, see: Boudjada (1980 ▶). For information about CuHPO_4_·0.5 H_2_O see: Sierra *et al.* (2003 ▶). For the structure of α-Cu_2_P_2_O_7_, see: Lukaszewicz (1966 ▶). For information about β-Cu_2_P_2_O_7_, see: Robertson & Calvo (1968 ▶). For a report about Cu_2_P_4_O_12_, see: Laügt *et al.* (1972 ▶).

## Experimental

### 

#### Crystal data

CuHPO_4_
*M* *_r_* = 159.52
Rhombohedral, 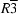

*a* = 9.5145 (4) Å
α = 114.678 (2)°
*V* = 495.88 (6) Å^3^
*Z* = 6
Mo *K*α radiation
μ = 6.92 mm^−1^
*T* = 183 K
0.05 × 0.03 × 0.03 mm

#### Data collection

Nonius KappaCCD diffractometer
Absorption correction: none
3338 measured reflections
755 independent reflections
654 reflections with *I* > 2σ(*I*)
*R*_int_ = 0.045

#### Refinement

*R*[*F* ^2^ > 2σ(*F* ^2^)] = 0.026
*wR*(*F* ^2^) = 0.066
*S* = 1.02
755 reflections
60 parameters
All H-atom parameters refined
Δρ_max_ = 0.62 e Å^−3^
Δρ_min_ = −0.66 e Å^−3^

### 

Data collection: *COLLECT* (Nonius, 1998 ▶); cell refinement: *DENZO* (Otwinowski & Minor 1997 ▶); data reduction: *DENZO*; program(s) used to solve structure: *SHELXS97* (Sheldrick, 2008 ▶); program(s) used to refine structure: *SHELXL97* (Sheldrick, 2008 ▶); molecular graphics: *DIAMOND* (Brandenburg, 2006 ▶); software used to prepare material for publication: *SHELXL97*.

## Supplementary Material

Crystal structure: contains datablocks I, global. DOI: 10.1107/S1600536809045632/fi2089sup1.cif

Structure factors: contains datablocks I. DOI: 10.1107/S1600536809045632/fi2089Isup2.hkl

Additional supplementary materials:  crystallographic information; 3D view; checkCIF report

## Figures and Tables

**Table 1:** Selected bond lengths (Å)

Cu1—O1	1.925 (2)
Cu1—O4^i^	1.932 (2)
Cu1—O3^ii^	1.971 (2)
Cu1—O3^iii^	1.992 (2)
Cu1—O4^iv^	2.360 (2)
P1—O4	1.515 (2)
P1—O1	1.530 (2)
P1—O3	1.541 (2)
P1—O2	1.571 (2)

Symmetry codes: (i) 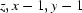; (ii) 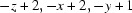; (iii) 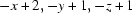; (iv) 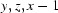.

**Table 2:** Hydrogen-bond geometry (Å, °)

*D*—H⋯*A*	*D*—H	H⋯*A*	*D*⋯*A*	*D*—H⋯*A*
O2—H2⋯O1^ii^	0.87 (5)	1.93 (5)	2.800 (3)	176 (5)

Symmetry code: (ii) 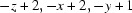.

**Fig. 1.**
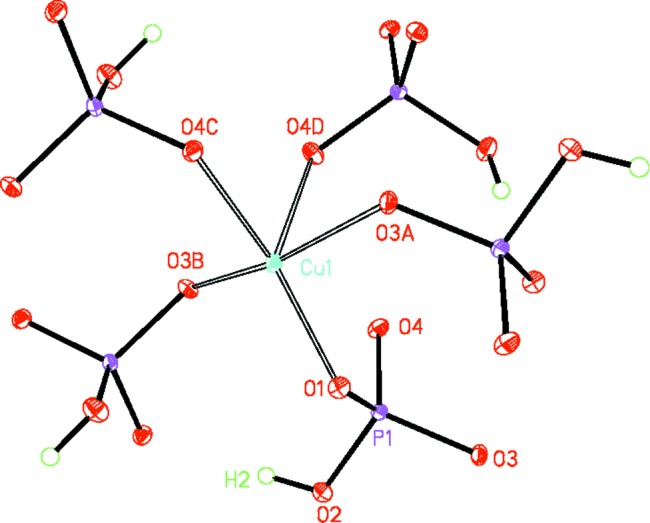
The molecular structure of 1, showing 50% probability displacement ellipsoides and the numbering scheme for the complete coordination polyhedron about Cu1 (Symmetry codes: (A) z,x-1,y-1; (B) -z+2,-x+2,-y+1; (C) -x+2,-y+1,-z+1 and (D) y,z,x-1.)

**Fig. 2.**
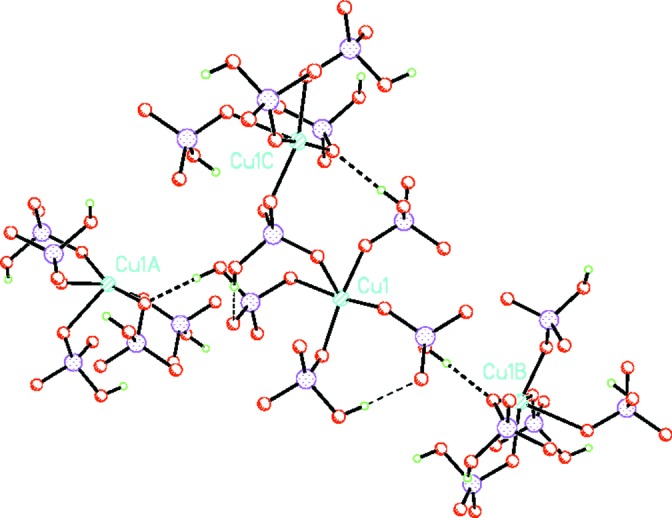
The intra and inter molecular O2—H2···O1 bonding about Cu1 (Symmetry codes: (A) z,x-1,y; (B) y+1,z,x-1 and (C) -y+1,-z+1,-x+1.)

**Fig. 3.**
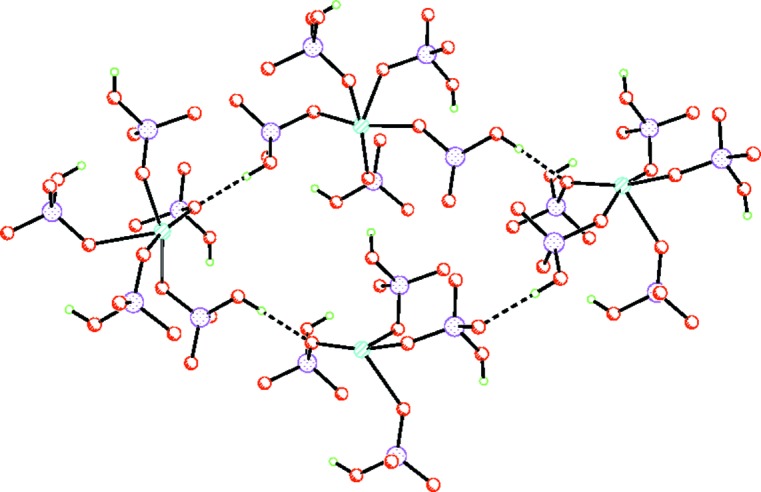
The intra and inter molecular O2—H2···O1 bonding about Cu1 (Symmetry codes: (A) z,x-1,y; (B) y+1,z,x-1 and (C) -y+1,-z+1,-x+1.)

**Fig. 4.**
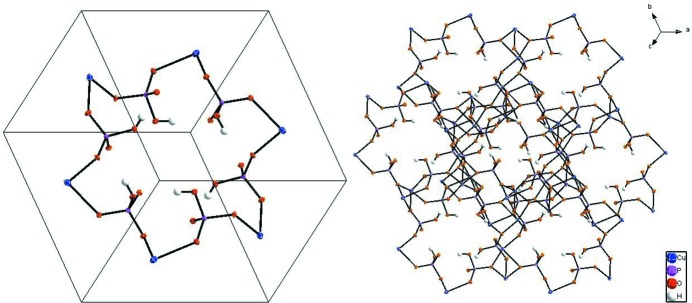
View of the unit cell of CuHPO~4~ along the z axis.

**Fig. 5.**
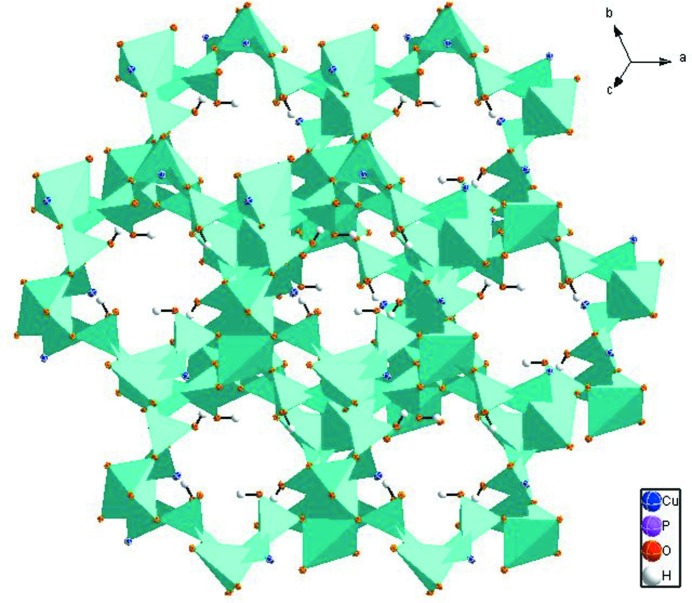
Projection of the CuHPO4 structure along the z axis, with applied polyhedrale.

## supplementary crystallographic information

### Comment

The hydrogen phosphate of copper(II) adopts the formula MHPO_4_ like other divalent cations. However, the monetites CaHPO_4_ (triclinic, *P*1; Smith *et al.*, 1955), BaHPO_4_ (orthorhombic, Pccn; Bengtsson, 1941) and PbHPO_4_ (monoclinic, P2/c or *Pc*; Bengtsson, 1941) or SrHPO_4_ (triclinic, *P*1; Boudjada *et al.*, 1978) and SnHPO_4_ (monoclinic, *P*2_1_/*c*; Berndt & Lamberg, 1971) have very different structures, which could be due to the much bigger ionic radii of the metals in comparison to copper. CuHPO_4_ has a rhombohedral (R3) structure. The coordination of Cu can be described as a square pyramid, with the apical C–O bond being significantly longer than the other four bonds. The coordination in the base plane could even be described as a strongly squeezed, almost planar tetrahedron (Fig 1). The Cu ions are linked by distorted PO_4_ tetrahedra yielding twelve-membered rings (see Fig. 4, and Fig. 5). The distortion of the phosphate tetrahedra is caused by the OH-groups, which point towards the centre of the rings. There is only one hydrogen bond present in the asymmetric unit (see hydrogen bond geometry). But in the whole crystal structure, this leads to two intramolecular and three intermolecular hydrogen bonds (see Fig. 2 and Fig. 3). In other copper phosphates, the copper atoms are coordinated by four, five and/or six oxygen atoms, respectively (Boudjada, 1980; Sierra *et al.*, 2003; Lukaszewicz, 1966; Robertson & Calvo, 1968; Laügt *et al.*, 1972). CuHPO_4_ formed only trigonal bipyramids of CuO_5_.

### Experimental

Phosphoric acid (65%) and copper oxide were mixed in a mortar for several hours. Afterwards the mixture was tempered at 373 K for a week. CuHPO_4_ was obtained in the form of emerald-green needles, which decompose by further tempering.

### Refinement

The hydrogen atom of the hydroxyd-group was located by difference Fourier synthesis and refined isotropically.

### Crystal data

**Table:**

CuHPO_4_	*D*_x_ = 3.205 Mg m^−^^3^
*M**_r_* = 159.52	Mo *K*α radiation, λ = 0.71073 Å
Rhombohedral, *R*3	Cell parameters from 3338 reflections
Hall symbol: -P 3*	θ = 3.4–27.5°
*a* = 9.5145 (4) Å	µ = 6.92 mm^−^^1^
α = 114.678 (2)°	*T* = 183 K
*V* = 495.88 (6) Å^3^	Needles, green
*Z* = 6	0.05 × 0.03 × 0.03 mm
*F*(000) = 462	

### Data collection

**Table:**

Bruker–Nonius KappaCCD diffractometer	654 reflections with *I* > 2σ(*I*)
Radiation source: fine-focus sealed tube	*R*_int_ = 0.045
graphite	θ_max_ = 27.5°, θ_min_ = 3.4°
φ and ω scans	*h* = −11→12
3338 measured reflections	*k* = −12→12
755 independent reflections	*l* = −12→12

### Refinement

**Table:**

Refinement on *F*^2^	Secondary atom site location: difference Fourier map
Least-squares matrix: full	Hydrogen site location: inferred from neighbouring sites
*R*[*F*^2^ > 2σ(*F*^2^)] = 0.026	All H-atom parameters refined
*wR*(*F*^2^) = 0.066	*w* = 1/[σ^2^(*F*_o_^2^) + (0.0403*P*)^2^ + 0.166*P*] where *P* = (*F*_o_^2^ + 2*F*_c_^2^)/3
*S* = 1.02	(Δ/σ)_max_ < 0.001
755 reflections	Δρ_max_ = 0.62 e Å^−^^3^
60 parameters	Δρ_min_ = −0.66 e Å^−^^3^
0 restraints	Extinction correction: *SHELXL97* (Sheldrick, 2008), Fc^*^=kFc[1+0.001xFc^2^λ^3^/sin(2θ)]^-1/4^
Primary atom site location: structure-invariant direct methods	Extinction coefficient: 0.014 (2)

### Special details

**Table:**

Geometry. All e.s.d.'s (except the e.s.d. in the dihedral angle between two l.s. planes) are estimated using the full covariance matrix. The cell e.s.d.'s are taken into account individually in the estimation of e.s.d.'s in distances, angles and torsion angles; correlations between e.s.d.'s in cell parameters are only used when they are defined by crystal symmetry. An approximate (isotropic) treatment of cell e.s.d.'s is used for estimating e.s.d.'s involving l.s. planes.
Refinement. Refinement of *F*^2^ against ALL reflections. The weighted *R*-factor *wR* and goodness of fit *S* are based on *F*^2^, conventional *R*-factors *R* are based on *F*, with *F* set to zero for negative *F*^2^. The threshold expression of *F*^2^ > σ(*F*^2^) is used only for calculating *R*-factors(gt) *etc*. and is not relevant to the choice of reflections for refinement. *R*-factors based on *F*^2^ are statistically about twice as large as those based on *F*, and *R*- factors based on ALL data will be even larger.

### Fractional atomic coordinates and isotropic or equivalent isotropic displacement parameters (Å^2^)

**Table:**

	*x*	*y*	*z*	*U*_iso_*/*U*_eq_	
Cu1	0.91592 (6)	0.43161 (6)	0.19005 (6)	0.00678 (18)	
P1	1.31526 (12)	0.74973 (12)	0.68353 (13)	0.0059 (2)	
O1	1.1314 (4)	0.5149 (4)	0.4494 (3)	0.0083 (5)	
O2	1.5471 (4)	0.8561 (4)	0.7726 (4)	0.0106 (5)	
O3	1.3326 (3)	0.7437 (3)	0.8490 (3)	0.0077 (5)	
O4	1.2753 (4)	0.8882 (3)	0.6838 (3)	0.0087 (5)	
H2	1.555 (8)	0.866 (8)	0.689 (8)	0.040 (14)*	

### Atomic displacement parameters (Å^2^)

**Table:**

	*U*^11^	*U*^22^	*U*^33^	*U*^12^	*U*^13^	*U*^23^
Cu1	0.0063 (2)	0.0080 (3)	0.0069 (2)	0.0048 (2)	0.0048 (2)	0.0059 (2)
P1	0.0063 (4)	0.0066 (4)	0.0068 (4)	0.0049 (3)	0.0050 (3)	0.0054 (3)
O1	0.0084 (11)	0.0069 (11)	0.0079 (11)	0.0053 (10)	0.0055 (10)	0.0050 (10)
O2	0.0096 (11)	0.0151 (12)	0.0120 (11)	0.0095 (10)	0.0086 (10)	0.0106 (10)
O3	0.0079 (10)	0.0090 (11)	0.0074 (10)	0.0056 (9)	0.0060 (9)	0.0063 (9)
O4	0.0109 (11)	0.0085 (10)	0.0070 (10)	0.0076 (9)	0.0059 (9)	0.0058 (9)

### Geometric parameters (Å, °)

**Table:**

Cu1—O1	1.925 (2)	P1—O3	1.541 (2)
Cu1—O4^i^	1.932 (2)	P1—O2	1.571 (2)
Cu1—O3^ii^	1.971 (2)	O2—H2	0.87 (5)
Cu1—O3^iii^	1.992 (2)	O3—Cu1^v^	1.971 (2)
Cu1—O4^iv^	2.360 (2)	O3—Cu1^iii^	1.992 (2)
P1—O4	1.515 (2)	O4—Cu1^vi^	1.932 (2)
P1—O1	1.530 (2)	O4—Cu1^vii^	2.360 (2)
			
O1—Cu1—O4^i^	163.91 (9)	O1—P1—O3	110.78 (12)
O1—Cu1—O3^ii^	91.59 (9)	O4—P1—O2	111.98 (13)
O4^i^—Cu1—O3^ii^	94.28 (9)	O1—P1—O2	109.68 (13)
O1—Cu1—O3^iii^	94.20 (9)	O3—P1—O2	102.64 (12)
O4^i^—Cu1—O3^iii^	84.72 (9)	P1—O1—Cu1	123.24 (13)
O3^ii^—Cu1—O3^iii^	162.12 (8)	P1—O2—H2	110 (3)
O1—Cu1—O4^iv^	112.90 (9)	P1—O3—Cu1^v^	128.15 (13)
O4^i^—Cu1—O4^iv^	83.13 (4)	P1—O3—Cu1^iii^	126.96 (13)
O3^ii^—Cu1—O4^iv^	74.64 (8)	Cu1^v^—O3—Cu1^iii^	101.63 (10)
O3^iii^—Cu1—O4^iv^	87.53 (8)	P1—O4—Cu1^vi^	132.92 (13)
O4—P1—O1	110.21 (12)	P1—O4—Cu1^vii^	125.51 (12)
O4—P1—O3	111.35 (12)	Cu1^vi^—O4—Cu1^vii^	90.84 (8)

Symmetry codes: (i) *z*, *x*−1, *y*−1; (ii) −*z*+2, −*x*+2, −*y*+1; (iii) −*x*+2, −*y*+1, −*z*+1; (iv) *y*, *z*, *x*−1; (v) −*y*+2, −*z*+1, −*x*+2; (vi) *y*+1, *z*+1, *x*; (vii) *z*+1, *x*, *y*.

### Hydrogen-bond geometry (Å, °)

**Table:**

*D*—H···*A*	*D*—H	H···*A*	*D*···*A*	*D*—H···*A*
O2—H2···O1^ii^	0.87 (5)	1.93 (5)	2.800 (3)	176 (5)

Symmetry codes: (ii) −*z*+2, −*x*+2, −*y*+1.
